# Ovarian steroid cell tumors, not otherwise specified: three case reports and literature review

**DOI:** 10.3389/fonc.2024.1400085

**Published:** 2024-07-04

**Authors:** Yue Sun, Lina Tian, Chao Meng, Guoyan Liu

**Affiliations:** ^1^ Department of Gynecology and Obstetrics, Tianjin Medical University General Hospital, Tianjin, China; ^2^ Tianjin Key Laboratory of Female Reproductive Health and Eugenics, Tianjin Medical University General Hospital, Tianjin, China; ^3^ Department of Gynecologic Oncology, Tianjin Medical University Cancer Institute and Hospital, Key Laboratory of Cancer Prevention and Therapy of Tianjin, National Clinical Research Center for Cancer, Tianjin’s Clinical Research Center for Cancer, Tianjin, China

**Keywords:** ovarian steroid cell tumors, not otherwise specified, virilization, testosterone, treatment

## Abstract

**Objective:**

To provide a reference for the diagnosis and treatment of ovarian steroid cell tumors, not otherwise specified (SCTs-NOS).

**Methods:**

We retrospectively analyzed the clinicopathological data of three patients with SCTs-NOS admitted to the Tianjin Medical University General Hospital from 2012 to 2022 and reviewed literature reports related to this disease.

**Results:**

A total of 3 cases in our center and 70 cases searched in literature reports were included. The age at diagnosis ranged from 3 to 93 years (median, 34 years). The common clinical manifestations were hirsutism, acne, deepened voice, clitoromegaly, amenorrhea, and excessive weight gain. Tumor sizes ranged from 1.2 to 45 cm, with an average diameter of 6.5cm. Most of SCTs-NOS were benign, but some of them exhibited malignant behavior. Surgery was the main treatment and close follow-up was required. The follow up time of 73 cases ranged from 3 to 132 months (median, 21.3 months). Disease recurrence or progression occurred in 14 cases (19.2%). Three of the 73 patients had a successful pregnancy.

**Conclusion:**

SCTs-NOS usually occur in women of reproductive age, which are mainly manifested as androgen excess symptoms. Surgery is an appropriate treatment for SCTs-NOS and should be individualized. Final diagnosis depends on pathology. SCTs-NOS have malignant potential, and the treatments for patients with malignant tumors and disease recurrence or progression were cytoreductive surgery, adjuvant chemotherapy, and gonadotrophin-releasing hormone agonists (GnRHa) therapy.

## Introduction

Ovarian steroid cell tumors (SCTs) are rare sex cord stromal tumors, accounting for less than 0.1% of all ovarian tumors (1). Based on the cell origin, they include three subtypes: stromal luteomas, Leydig cell tumors, and not otherwise specified (NOS). SCTs-NOS usually occur in women of childbearing age, with an average age of 43 years (2). The major presenting symptoms of SCTs-NOS are hirsutism, acne, deepened voice, clitoromegaly, amenorrhea, Cushing’s syndrome, and infertility, caused by excessive secretion of steroid hormones. SCTs-NOS account for about 50-60% of SCTs and are usually benign, but some of them do exhibit malignant behavior (3, 4). Surgery is an appropriate treatment for SCTs-NOS, which should be individualized according to age, surgical stage, tumor histopathology, and fertility desires (5). There has been limited data on SCTs-NOS regarding the clinicopathological characteristics, therapy and prognosis now. In this article, we retrospectively analyzed the clinicopathological data of three patients with SCTs-NOS admitted to the Tianjin Medical University General Hospital from 2012 to 2022 and reviewed literature reports related to this disease to explore the clinical manifestations, imaging findings, histopathological and immunohistochemical features, treatment and prognosis of SCTs-NOS, so as to deepen clinicians’ understanding of this disease, increase the preoperative diagnosis rate and improve prognosis.

## Case report

**Figure 1:**
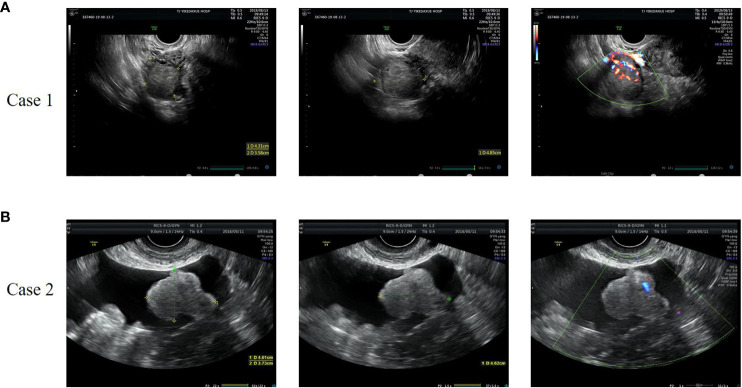
Representative images of gynecologic ultrasound of case 1 **(A)** and case 2 **(B)**. **(A)** The ultrasound showed a 4.3×4.9×3.6cm low-moderate echo mass on the right side of the uterus with rich blood flow signals. **(B)** The ultrasound revealed a solid hypoechoic lobulated mass in the left adnexal area, with a size of 5.6×4.6×3.7cm and striated blood flow signal.

**Figure 2:**
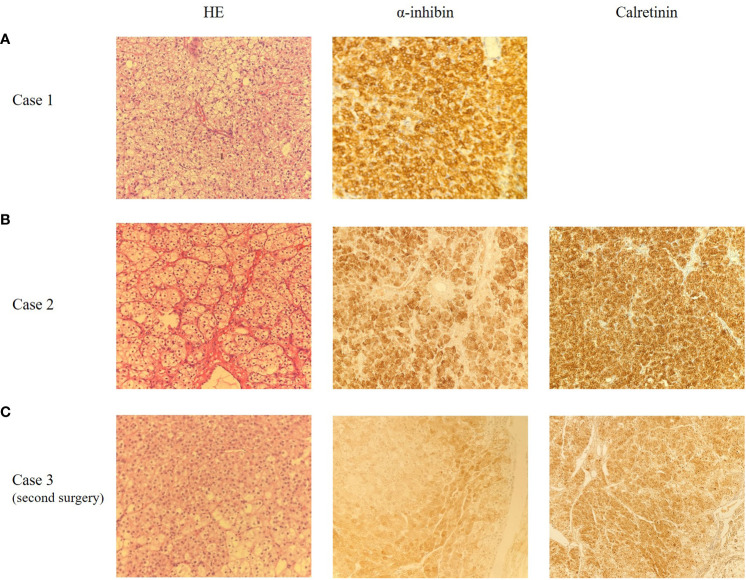
Representative microscopic appearance of case 1 **(A)**, case 2 **(B)** and case 3 **(C)**, magnification 200X. **(A)** The pathological report indicated ovarian SCTs-NOS with poorly defined local tumor boundaries. Immunohistochemical staining for α-inhibin was positive. **(B)** The pathological report indicated ovarian SCTs-NOS. Immunohistochemical staining for α-inhibin and Calretinin were positive. **(C)** The pathological report indicated mesenteric nodules SCTs-NOS with bleeding, necrosis and calcification. Immunohistochemical staining for α-inhibin and Calretinin were positive.

**Case 1:** A 37-year-old woman was referred to our hospital because of amenorrhea for 6 months and pelvic mass for 1 month. She was an overweight female with a body mass index (BMI) of 28.6kg/m^2^ and had a normal blood pressure. Gynecological examination revealed normal vulva development, clitoris hypertrophy, and a 4×4cm solid mass in the right adnexal area. Laboratory tests showed a significant increase in serum total testosterone (363.73ng/dL, normal range, 10.83~56.94 ng/dL). Tumor markers (AFP 1.75ng/ml, CEA 0.73 ng/ml, CA199 4.16U/mL, CA125 13.20U/mL, HE4 26.35pmol/L, HCG<1.20mIU/mL), thyroid function, insulin release test and adrenal function were within normal ranges. Transvaginal ultrasound showed a 4.3×4.9×3.6cm low-moderate echo mass on the right side of the uterus with rich blood flow signals (**Figure 1A**). A laparoscopic right salpingo-oophorectomy was then performed. Intraoperative exploration revealed a right ovarian mass with a size of 5×5×5cm and a smooth surface. The cut sections of the sample were golden yellow, solid and fine in texture. Postoperative pathological report was: (right ovary) SCTs with poorly defined local tumor boundaries (**Figure 2A**). Immunohistochemical staining results showed α-inhibin (+), Calretinin (+), Cytokeratin (CK) local (+), epithelial membrane antigen (EMA) (–) and a Ki-67index of 5%. Combined with immunohistochemical results and clinical signs, this was consistent with ovarian sex cord stromal tumors, tend to be NOS. Testosterone was 31.24ng/dL at 10 days after surgery. Her menstruation resumed 1 month after the surgery.

**Case 2**: A 58-year-old female patient was admitted to our hospital because of pelvic mass for 10 days during a routine gynecologic ultrasound without any symptoms of discomfort. The patient underwent modified radical mastectomy of the left breast due to invasive ductal carcinoma of the breast 8 years ago and received 6 courses of chemotherapy (doxorubicin, cyclophosphamide, and docetaxel) after surgery. The patient was regularly reviewed with mammography, and no abnormal manifestations were found. The patient also underwent partial nephrectomy due to clear cell carcinoma of the left kidney 1 year ago, and no abnormal findings were found in regular follow-up. Her BMI was 25.7kg/m^2^ and her blood pressure was normal. The characteristics of vulva were consistent with those of postmenopausal women. The left adnexal area was thickened with tenderness on palpation. Serum testosterone was elevated, with a value of 240.8ng/dL. The tumor markers were as follows: AFP 1.60ng/ml, CEA 0.92 ng/ml, CA199 6.67U/mL, CA125 11.9U/mL, HE4 27.32pmol/L. No significant abnormalities were found in other laboratory findings. Gynecologic ultrasound revealed a solid hypoechoic lobulated mass in the left adnexal area, with a size of 5.6×4.6×3.7cm, and ascites (**Figure 1B**). Abdominal computed tomography (CT) in other hospital showed pelvic effusion, and no abnormal density shadows were observed in bilateral adnexal areas. Pelvic magnetic resonance imaging (MRI) in other hospital revealed a solid mass in the left adnexal area, 5.3×6.2×5.9cm in size, and pelvic effusion, which suggested a high possibility of metastasis. Positron Emission Tomography-Computed Tomography (PET-CT) reported a low-density mass in the left adnexa with FDG isometabolism and ACE high metabolism. This patient subsequently underwent laparoscopic surgery. During the operation, a solid mass was found in the left ovary, about 4×3×3cm in size, with a yellow brown color, a gyriform and smooth surface, and abundant nutrient vessels. The left adnexa was resected and sent for a frozen section. The results showed that metastatic clear cell renal cell carcinoma was considered in the left ovary, and SCT was not excluded. According to the pathological findings, total hysterectomy and right salpingo-oophorectomy were continued performed. Postoperative paraffin pathological report revealed: (left ovary) SCT (**Figure 2B**). Immunohistochemical results showed that Calretinin, α-inhibin and Vimentin were positive, CD10, WMA and RCC were negative, which was consistent with SCTs-NOS. Serum testosterone was decreased to normal levels after surgery, and no recurrence of disease has been found for 42 months post-surgery.

**Case 3**: A 31-year-old woman was admitted to hospital for hirsutism for 1 year, abdominal distension and frequent urination for 2 months. She had a regular menstruation. She had excessive hair started at the age of 30, mainly on the chin and both lower limbs, and gradually worsened over several months, requiring weekly chin shaving. A physical examination revealed a BMI of 27.9 kg/m^2^, indicating an overweight woman, and elevated blood pressure (BP 155/120mmHg). Lots of coarse hair appeared on her chin, lower abdomen, and both lower limbs. She also had acanthosis nigricans on the back of her neck and armpits, but lacked acne, deepening voice, and other clinical features of Cushing’s syndrome. Gynecological examination showed that pubic hair was increased and distributed in a masculine way. A 15×15×10cm massive mass with clear boundaries was palpated in the lower abdomen, reaching 2 fingers above the navel. Serum testosterone levels were increased to 252ng/dL. The value of CA125 is 30.1U/mL. Gynecologic ultrasound showed that a 14.3×9×13.4cm hypoechoic mass could be seen above the uterus, which was closely related to the uterus, and part of the liquid dark area and dotted blood flow signal could be seen in it. This patient first underwent a left salpingo-oophorectomy. Intraoperative frozen section revealed a left ovarian thecoma with necrosis, so the scope of operation was not expanded. Postoperative pathological findings showed SCTs-NOS of the left ovary. Blood pressure and serum testosterone dropped to normal levels on the first day after surgery and hair on the face and limbs began to fall off. Hirsutism and virilization recurred 45 months after surgery, and disease recurrence was considered. Blood pressure (160-195/120-100mmHg) and serum testosterone (100ng/dL) levels were increased again. Abdominal enhanced CT showed multiple masses and nodules on both sides of the abdominal aorta and multiple nodules with deficient blood supply in the liver, which were considered metastatic tumors. The PET-CT report demonstrated multiple low-density masses and nodules in retroperitoneum with high metabolism and multiple intrahepatic low-density foci, which also indicated tumor recurrence or metastasis. After a multi-disciplinary treatment, the patient underwent an exploratory laparotomy. During the operation, multiple convex masses were observed on retroperitoneum, para-aorta and liver surface, with a maximum diameter of about 13cm. The masses were densely adherent to the mesentery surface, and the para-aortic lymph nodes are enlarged. Retroperitoneal mass resection + cholecystectomy + partial hepatectomy + para-aortic lesion resection + para-aortic lymph node dissection + greater omentectomy were then performed. The postoperative pathological results revealed mesenteric nodules SCT with bleeding, necrosis and calcification, which is considered malignant in combination with clinical and growth pattern. There were metastasis in the liver and upper paraaortic lymph nodes (**Figure 2C**). Immunohistochemical results indicated that Calretinin, CD99 and α-inhibin were diffusively positive, CK and EMA were partially positive, and Vimentin was negative. These findings suggested recurrence of malignant SCTs-NOS with retroperitoneal and hepatic metastasis. Serum testosterone decreased to 32.00ng/dL one day after surgery. Bleomycin + etoposide + cisplatin (BEP) chemotherapy combined with gonadotropin-releasing hormone agonist (GnRHa) adjuvant therapy was administered 13 days after surgery for a total of 6 courses. The patient relapsed again 9 months and died 12 months after the second operation.

## Discussion

SCTs-NOS are uncommon ovarian sex cord stromal tumors, the etiology and pathogenesis of which remain unclear. Apart from the investigation of 63 cases by Hayes and Scully (5), there has been limited data on SCTs-NOS regarding clinical retrospective studies now. Considering the rarity of the disease, we reported the treatment experience of 3 patients with SCTs-NOS in our center (**Table 1**) and summarized 70 cases reported in the literature from 1998 to 2023 (**Table 2**), with a view to providing recommendations for the diagnosis and treatment of the disease. Our retrospective study did not collect information on the 63 cases in Hayes and Scully’s article because of the lack of detail in each case. We also excluded some cases due to insufficient follow-up data or too short follow-up (less than 3 months).

**Table 1:** Clinicopathological features of three cases reported in our hospital.

	Case 1	Case 2	Case 3
Age (years)	37	58	31
Symptoms	Amenorrhea	No symptoms	Hirsutism, virilization, abdominal distension, frequent urination
Hypertension	No	No	Yes
BMI (kg/m^2^)	28.6	25.7	27.9
Testosterone (ng/dL)	363.7	240.8	252.0
Tumor diameter (cm)	4.9	5.6	14.3
Tumor side	Unilateral	Unilateral	Unilateral
Gynecologic ultrasound			
texture	Solid	Solid	Solid
form	Ellipsoid	Lobulate	Lobulate
echo	Low-moderate echo	Hypoechoic	Hypoechoic
CDFI	Rich blood flow signal	Striated blood flow signal	Dotted blood flow signal
Immunohistochemical results			
Calretinin	(+)	(+)	(+)
α-inhibin	(+)	(+)	(+)
CK	Local (+)	/	Partial (+)
EMA	(-)	/	Partial (+)
Vimentin	/	(+)	(-)
Therapy	RSO	HE, BSO	LSO; Retroperitoneal mass resection+cholecystectomy+ partial hepatectomy+para-aortic lesion resection+para-aortic lymph node dissection+greater omentectomy were then performed 45 months later, and BEP chemotherapy combined with GnRH treatment was performed for 6 months.
Follow-up (months)	No recurrence (39)	No recurrence (42)	Recurred (45) and died (57)
Pregnancy outcome	No pregnancy	No pregnancy	No pregnancy

LSO, left salpingo-oophorectomy; RSO, right salpingo-oophorectomy; BSO, bilateral salpingo-oophorectomy; HE, hysterectomy; BEP, bleomycin + etoposide + cisplatin; GnRHa, gonadotrophin-releasing hormone agonists. “-”, negative; “+”, positive; “/”, not applicable.

**Table 2:** Clinicopathological features of cases reported in the literature.

Case	Author	Age	BMI (kg/m^2^)	Surgery	Diameter	Pathological features				Adjuvant therapy	Follow-up (months)	Pregnancy outcome
						Necrosis	Hemorrhage	Mitosis (≥2 per 10 HPF)	Nuclear atypia (grade II/III)			
1	Wang (1998) (6)	48	/	HE,RSO,PLND,OE	4	–	–	–	+	–	No recurrence (6)	/
2	Faraj (1998) (7)	56	/	BO	4.5	/	/	/	/	–	No recurrence (12)	/
3	Brewer (1998) (8)	58	/	HE, BSO,TD	10	/	/	+(9)	+	BEP×2, GnRHa	Recurred (28)	/
4	Reedy (1999) (9)	46	/	HE,LSO,PLNS,OE	3.6	–	–	–	–	–	No recurrence (12)	/
5	Bas (2000) (10)	9	/	HE,BSO	2.5	–	–	–	–	Glucocorticoid	No recurrence (52)	/
6	Powell (2000) (11)	93	/	HE, BSO, sigmoid-ectomy,colostomy	21	+	/	+	+	–	Recurred and multi metastasis (10)	/
7	Stephens (2002) (12)	67	/	HE,BSO	1.2	–	–	–	–	–	No recurrence (3)	/
8	Garduno (2002) (13)	34	28.0	RSO	/	/	/	/	/	/	Recurred (132)	/
9	Liu (2005) (14)	30	>28.0	Tumor resection	3	–	–	–	–	–	No recurrence (12)	/
10	Kim (2007) (15)	52	>28.0	BSO, PLNS, OE	7.5	–	–	–	–	–	No recurrence (24)	/
11	Ding (2007) (16)	16	36.6	Tumor resection	5	/	/	/	/	/	No recurrence (36)	/
12	Gupta (2008) (17)	5	30.1	Tumor resection	4.5	/	/	/	/	–	No recurrence (4)	/
13	Stephens (2008) (18)	35	/	RSO	5	/	/	–	/	–	No recurrence (6)	/
14	Sawathiparnich (2009) (19)	6	20.9	LSO	7	+	–	+(2-5)	–	–	No recurrence (6)	/
15	Lee (2011) (20)	8	18.9	RO	5	–	–	–	–	–	No recurrence (16)	/
16	Zhang (2011) (21)	21	/	LSO, OE	5.8	–	–	–	–	–	No recurrence (3)	/
17	Varras (2011) (22)	40	>28.0	HE, BSO	6.8	–	–	–	–	–	No recurrence (18)	/
18	Layla (2011) (23)	65	/	BSO, TD	7	–	/	+(2)	–	BEP×6	Continued (18), lost to follow up	/
19	Arora (2012) (24)	22	/	HE, LSO	2	/	/	/	/	–	Recurred (108)	/
20	Singh (2012) (25)	70	>28.0	RO	5	/	/	/	/	–	No recurrence (5)	/
21	Yilmaz (2013) (26)	13	/	Tumor resection	2.5	–	–	–	–	–	No recurrence (6)	/
22	Sielert (2013) (27)	20	29.1	RO	5.5	–	–	–	–	–	No recurrence (18)	Uncomplicated pregnancy; female infant, 3,579 g at 40 weeks gestation
23	Jiang (2013) (28)	21	/	HE, BSO, OE	L 20, R 15	+	+	+ (>10)	+	PVB×4	Continued and died (10)	/
24	Jiang (2013) (28)	23	Normal	Tumor resection	6	–	–	–	–	–	No recurrence (36)	/
25	Swain (2013) (29)	28	/	LSO	5.9	/	/	/	/	–	No recurrence (12)	/
26	Chun (2013) (30)	52	/	LSO	6	–	–	+(5)	–	–	No recurrence (21)	/
27	Cooray (2013) (31)	54	/	HE, BSO	L 3, R 4	–	–	–	–	–	No recurrence (12)	/
28	Menghua (2014) (32)	28	/	LO	2.5	+	/	Active cell growth	/	–	Recurred (42), died(58)	/
29	Li (2014) (33)	29	/	RSO	6	+	+	+	+	Docetaxel + Nedaplatin ×8	Recurred (18), died (24)	/
30	Tai (2014) (34)	29	/	RSO	10	–	+	–	–	–	No recurrence (24)	/
31	Chung (2014) (35)	35	29.5	HE, BSO, PLND, OE, AE	4.9	–	–	–	–	GnRHa ×6	No recurrence (43)	/
32	Kim (2014) (36)	46	/	HE, BSO, PLND, OE	7.7	/	/	/	/	–	Recurred (60)	/
33	Haroon (2015) (37)	3	/	LSO	7	–	–	–	–	–	No recurrence (25)	/
34	Haroon (2015) (37)	26	/	RSO	4.5	–	–	–	–	–	No recurrence (15)	/
35	Haroon (2015) (37)	28	/	HE, BSO	3&2.5	–	–	+(2)	–	–	No recurrence (49)	/
36	Haroon (2015) (37)	32	/	HE, BSO	4	–	–	–	–	–	No recurrence (71)	/
37	Haroon (2015) (37)	37	/	LSO	7	–	–	–	–	–	No recurrence (23)	/
38	Haroon (2015) (37)	43	/	RSO	10	–	–	+(5)	+	–	Recurred (34)	/
39	Haroon (2015) (37)	50	/	RSO	7	–	–	–	–	–	No recurrence (11)	/
40	Haroon (2015) (37)	51	/	HE, BSO, PLND, OE	13	+	+	+(8)	+	–	Recurred and died (8)	/
41	Haroon (2015) (37)	52	/	LSO	9	–	–	+(4)	+	PVB ×4 & radioth-erapy	No recurrence (43)	/
42	Haroon (2015) (37)	67	/	BSO	3.4	–	–	–	–	–	No recurrence (27)	/
43	Yokozawa (2015) (38)	16	/	Tumor resection	4	–	–	–	–	–	No recurrence (15)	/
44	Ben (2015) (39)	39	/	LSO	3.4	/	/	/	/	–	No recurrence (8)	/
45	Qian (2016) (40)	5	/	RSO	8	–	–	–	–	–	No recurrence (60)	/
46	Santo (2016) (41)	21	/	RSO	11.5	–	+	+(4)	–	–	No recurrence (43)	/
47	Lee (2016) (42)	47	/	HE, BSO, OE	11	–	+	+(5)	+	BEP×3	No recurrence (24)	/
48	Sedhom (2016) (43)	67	/	HE, BSO, TD	9.8	/	+	+(22)	–	/	Died after operation	/
49	Zang (2017) (44)	46	/	LSO	12	/	/	/	–	–	No recurrence (12)	/
50	Chen (2018) (45)	40	/	RSO	6	/	/	/	/	–	No recurrence (36)	/
51	Yuan (2019) (46)	17	/	Tumor resection	8	–	–	–	–	–	No recurrence (6)	/
52	Wong (2019) (47)	24	26.0	RSO	4.5	–	–	–	–	–	No recurrence (9)	/
53	Alves (2019) (4)	30	Normal	Tumor resection	2	–	–	–	–	–	No recurrence (36)	/
54	Matsukawa (2019) (48)	22	37.7	LO	3	–	–	–	–	–	No recurrence (60)	/
55	Nakasone (2019) (49)	50	/	HE, BSO, PLNS	9	–	–	/	–	–	Recurred (70)	/
56	Yoshimatsu (2020) (50)	4	/	RSO	8	+	+	+(3)	–	BEP×3	No recurrence (7)	/
57	Faten (2020) (51)	36	/	RO	4.5	–	–	–	–	–	No recurrence (12)	/
58	Uyanıkoglu (2020) (52)	34	/	Tumor resection	4	–	–	–	–	–	No recurrence (13+)	Healthy infant at term
59	Schnuckle (2021) (53)	14	/	LSO	3.2	–	+	–	–	–	No recurrence (24)	/
60	Ismail (2021) (54)	21	19.1	LSO	15	–	–	–	–	–	No recurrence (3)	/
61	Lin (2022) (55)	14	30.1	OTR	<7	–	–	–	–	–	No recurrence (5)	/
62	Lin (2022) (55)	53	23.8	RSO	<7	–	–	–	–	–	No recurrence (9)	/
63	Lin (2022) (55)	25	29.6	OTR	<7	–	–	–	–	–	No recurrence (11)	/
64	Lin (2022) (55)	55	24.1	HE, BSO, OE,PLND	<7	–	–	–	–	–	No recurrence (12)	/
65	Lin (2022) (55)	51	27.9	HE, BSO	/	/	/	/	/	/	No recurrence (27)	/
66	Lin (2022) (55)	59	19.4	BSO	<7	–	–	–	–	–	No recurrence (28)	/
67	Lin (2022) (55)	43	22.2	HE, BS,OER	<7	–	–	–	–	–	No recurrence (32)	/
68	Lin (2022) (55)	68	24.8	EHE,BSO	<7	–	+	–	+	–	No recurrence (46)	/
69	Lin (2022) (55)	9	13.7	OTR	<7	–	–	–	+	–	No recurrence (53)	/
70	Matemanosak (2023) (56)	31	35.6	LSO	6	–	–	–	–	–	No recurrence (21.5)	Uncomplicated pregnancy; male infant, 3,495 g at 38 weeks gestation

LSO, left salpingo-oophorectomy; RSO, right salpingo-oophorectomy; BSO, bilateral salpingo-oophorectomy; LO, left oophorectomy; RO, right oophorectomy; BO, bilateral oophorectomy; HE, hysterectomy; OE, omentectomy; PLND, pelvic lymph node dissection; PLNS, pelvic lymph node sampling; AE, appendectomy; TD, tumor debulking; BEP, bleomycin + etoposide + cisplatin; PVB, cisplatin + vincristine + bleomycin. “-”, no; “+”, yes; “/”, not applicable.

SCTs-NOS can occur at any age but they are usually occur in women of reproductive age. The 73 patients with SCTs-NOS ranged in age from 3 to 93 years (median, 34 years). Clinical symptoms usually vary depending on hormonal secretion and tumor progression. SCTs-NOS can secrete many hormones, such as testosterone, estradiol, cortisol, and, rarely, prorenin (5). According to previous studies and our review, more than half (56-77%) of SCTs-NOS patients exhibit hyperandrogenic symptoms and signs of virilization, including hirsutism, acne, deep voice, clitoromegaly, amenorrhea, and infertility (6, 28, 40). 67% (20/30) of patients were overweight or obese. In addition, about 6-23% of women present with estrogenic manifestations, such as isosexual precocious puberty, abnormal uterine bleeding or postmenopausal bleeding (2, 5). Some patients even progress to endometrial cancer (57). About 6–10% of patients may have symptoms of Cushing’s syndrome because of elevated plasma cortisol levels (58, 59). Hypertension and hypokalemia may also, in rare cases, be unique manifestations in cases of increased serum prorenin levels from SCTs (60). However, approximately 25% of cases have other unspecific symptoms such as abdominal pain and distention, and are even asymptomatic (6, 30). In these cases, the diagnosis is usually made after postoperative histopathological confirmation.

Elevated testosterone and androstenedione levels are usually showed in serum laboratory tests, indicating an ovarian-derived androgen release and normal dehydroepiandrosterone sulfate (DHEA-S) levels, thus ruling out hyperandrogenemia caused by adrenal tumors (46). Elevated testosterone was found in all three cases we reported. Currently, there are no specific tumor markers for preoperative diagnosis of SCTs-NOS. Serum levels of CA125 and HE4 are common tumor markers in epithelial ovarian tumors (61), while they are generally maintained in the normal range in SCTs-NOS. And the literature data do not suggest whether increased levels indicate malignant potential (30, 51). This is relevant to the cases described because these tumor markers remain within the normal range even after the disease has recurred.

Imaging examinations are helpful in the evaluation of gynecological diseases. Gynecologic ultrasound is often used for the initial assessment of ovarian tumors. SCTs-NOS usually occur in the unilateral ovary, and grayscale ultrasound often shows solid, round or oval nodules with clear boundaries and mainly slightly hypoechoic internal echoes. The intensity of the echo might be relevant to the internal fat content. Due to the increased vasodilator effect of testosterone, most SCTs-NOS exhibit rich blood flow signals dominated by low resistance blood flow (42, 62). Altogether, the more typical ultrasound imaging features of SCTs-NOS are round or oval, solid, slightly hypoechoic masses with uniform internal echo, abundant blood supply and low resistance, which is consistent with the three cases we reported. However, gynecologic ultrasound has a limited sensitivity in detecting SCTs-NOS. MRI is effective in differentiating epithelial from non-epithelial ovarian tumors, including SCTs-NOS. The MRI features of SCTs-NOS depend on the amount of lipid components and fibrous stroma in the tumor, which mainly appears as solid masses with equal or slightly high signal on T1-weighted images, high signal on T2-weighted images, and high signal on diffusion weighted images (63). In addition, the lesions are significantly enhanced after treatment with gadolinium-diethylenetriamine pentaacetic acid (Gd-DTPA) (55). MRI imaging findings indicative of epithelial ovarian cancers include solid or cystic-solid soft tissue mass, lobed or irregular in shape, blurred boundaries, intracystic papillary projections, equal and low T1 signal intensity and high T2 signal intensity. Granulosa cell tumors (GCTs) are usually present as large, multilocular masses with both solid and cystic components and areas of hemorrhage. Their most typical appearance is a solid mass with a spongelike (“Swiss cheese”) appearance; the tumor’s cystic compartments may be hemorrhagic fluid with high T1 signal and fluid-fluid levels. Liposarcomas are the most common type of retroperitoneal sarcomas. Increased signal intensity on T1-weighted images and signal loss after fat saturation is indicative of a fat-containing lesion. If a fatty mass presents with irregular and ill-defined borders, then the diagnosis of liposarcoma should be considered (64). On the other hand, the role of CT in revealing cystic lesions and lipid components may be limited (65, 66). Abdominal CT may reveal a moderate heterogeneous enhancement on the ovary (67). Although clinical and radiological examinations provide useful clues for establishing the initial differential diagnosis of ovarian tumors, diagnosing SCTs-NOS remains challenging, and surgery can be used for both diagnosis and definitive treatment.

Currently, there are no established treatment options due to the low incidence of SCTs-NOS. The treatment of the tumor depends on several factors, including the age, tumor stage, tumor histopathology, presence of malignant features, and fertility desires. Surgical resection is considered the primary option for benign SCTs-NOS. Therefore, unilateral salpingooophorectomy or tumor removal is recommended for women who have fertility desire and early-stage disease (46), as in our case 1 and 3. Serum testosterone levels should be measured with regular follow-up after surgery. On the other hand, total hysterectomy and bilateralsalpingo-oophorectomy are mostly performed in postmenopausal women and those who have no fertility desire (28, 40). Because many patients may also have endometrial hyperplasia or even endometrial cancer, endometrial sampling should be performed when planning fertility preserving surgery, which may influence the decision to perform a hysterectomy.

Certainly, histopathological examination is the gold standard for the final diagnosis of SCTs-NOS (68). SCTs-NOS are unilateral in 94% of cases, and typically solid and well-circumscribed. Their sizes range from 1.2 to 45 cm, with an average diameter of 6.5cm in the 76 cases we summarized. Microscopically, SCTs are composed of large polygonal cells with vacuolar cytoplasm and smaller cells with abundant granular eosinophilic cytoplasm. These cells are usually arranged diffusely or in small nests within the vascular stroma. The absence of cytoplasmic Reinke crystals is useful to differentiate SCTs-NOS from Leydig cell tumors. Besides, Leydig cell tumors are usually located in hilar locations, and commonly correlated with Leydig cell hyperplasia (1, 48). Stromal luteomas, typically characterized by stromal hyperplasia with degenerative pseudovascular spaces, are confined to the ovarian stroma and often accompanied by stromal hyperthecosis (69). SCTs-NOS may have fibromatous components, similar to those of thecomas, but these components account for less than 10% of the tumor (70). The absence of spindle cells and fibromatous background help to distinguish them from luteinized thecomas. Pregnancy luteomas are mostly multifocal (bilateral in 1/3 cases), usually detected during cesarean section, and resolve spontaneously after pregnancy (69). Besides, both primary and metastatic ovarian clear cell carcinomas can be ruled out by the lack of glycogen-rich cytoplasm and eccentric nuclei (40, 68, 71).

Apart from microscopic appearances, immunohistochemistry is also extremely helpful for correct diagnosis. α-inhibin and Calretinin are considered to be the most useful markers in the differential diagnosis of SCTs-NOS. The sensitivity of α-inhibin reactivity is 5% to 90%, whereas the sensitivity of positive Calretinin ranges from 60% to 90% (3, 53). α-inhibin and Calretinin were positively expressed in all three patients at our center. SCTs-NOS are also commonly positive for Vimentin, Melan-A, and CD99, and variably positive for AE1/3, CAM5.2, HMB45, and S100 (3). Currently, techniques of steroidogenic enzymes immunohistochemical staining are being developed to identify SCTs-NOS. Ovarian steroidogenic enzymes, including 3 β-HSD, P450c17, 5 α-reductase 1/2, 17 β HSD 5, and 17 β HSD 1, are obviously expressed in the SCTs-NOS tumor cells. However, no adrenal-specific steroidogenic enzymes such as P450c21, DHEA-ST, and CYP11B1/2 are found to be expressed in SCTs-NOS, indicating that adrenocortical hormones are not synthesized (48). Validated enzymes may be used as markers for differential diagnosis of hyperandrogenic ovarian disease in the future.

Usually, SCTs-NOS are benign, but 25%-43% of SCTs-NOS have malignant potential, with metastasis beyond the ovaries found in 20% of cases. Hayes and Scully summarized five pathological features associated correlated with malignant potential in their studies, including the presence of more than two mitotic figures per 10 high power fields in 92% of cases, necrosis in 86% of studies, tumor sizes larger than 7cm in 78%, hemorrhage in 77%, and grade 2–3 atypia in 64% of cases respectively (5). Although some patients may have clinical malignant features such as massive ascites, metastasis, and satellite nodules, confirming malignancy must be determined according to the above pathological features. Of the 70 cases retrieved, about half (30/57) presented with at least one malignant feature, except for 13 cases with missing pathological details. After primary surgery, 13 patients relapsed and 9 patients had at least two malignant pathological features. In our three cases, no malignant pathological features were found after the initial surgery, but three malignant pathological features were observed in specimens resected after recurrence and metastasis in the third case.

As SCTs-NOS have malignant potential, postoperative follow-up is required, the optimal duration of follow-up has not been determined due to the limited understanding of the mechanisms of tumorigenesis of these tumors. The follow up time of 73 cases ranged from 3 to 132 months, with a median time of 21.3 months. Disease recurrence or progression occurred in 14 cases (19.2%) (**Tables 1**, **2**). The reported treatments for patients with malignant tumors and disease recurrence or progression in SCTs-NOS were cytoreductive surgery, adjuvant chemotherapy, and GnRHa therapy. The current possible adjuvant chemotherapy regiments are as follows: BEP (bleomycin, etoposide, and cisplatin); PVB (cisplatin, vinblastine, and bleomycin); cisplatin, doxorubicin, and cyclophosphamide; taxane and platinum; and bleomycin, vinblastine, and cisplatin (50, 72–75). In a patient with SCTs-NOS who reported recurrence 5 years after the initial surgery, intraperitoneal spread and liver metastases were completely removed by reduction surgery, radiofrequency ablation of liver metastasis, and adjuvant BEP (36). In our review of the literature, seven patients received adjuvant chemotherapy, of which four received the BEP chemotherapy regimen (**Table 2**). In two reported cases, tumor progression did not respond to chemotherapy alone. Another relapsed case did not respond to chemotherapy, but was controlled after switching to GnRHa treatment and remained tumor-free for 22 months. Pascale previously found that androgen secretion by ovarian virilizing tumors may be gonadotrophin dependent, and GnRHa may exert an inhibitory effect by reducing hormone secretion and inducing apoptosis of tumor cells (76). Wang also demonstrated that the serum testosterone level was still elevated after surgery in a patient whose tumor was completely removed during surgery. After one cycle of GnRHa treatment, the patient’s blood testosterone returned to normal levels, and there was no sign of recurrence during the subsequent 32 months of follow-up (77). Those findings suggest that GnRHa plays an important role in relapsed SCTs-NOS patients. When multidrug chemotherapy fails, some patients may exhibit a response to subsequent treatment with GnRHa (78). However, treatment decisions remain controversial and more research is needed to discuss and confirm the best treatment options.

Since SCTs-NOS mostly occur in women of childbearing age, more attention should be paid to the protection of ovarian function and reproductive outcomes. Data on fertility outcomes after surgery for SCTs-NOS are limited. As far as we know, only three cases of spontaneously conceived pregnancies with good outcomes have been reported (**Table 2**). None of the three patients we reported had a subsequent pregnancy.

## Conclusion

SCTs-NOS are rare, and thus it is difficult to conduct large-scale clinical cohort studies and there is a lack of consistent treatment recommendations. SCTs-NOS can occur in women of all ages and cause symptoms such as virilization, isosexual precocious puberty in adolescents, and Cushing’s syndrome. Although endocrine abnormalities usually recover after surgery, the resulting developmental effects remain, especially in adolescents. Therefore, it is of great significance to improve the early diagnosis of the disease and decrease the developmental abnormalities in young patients due to endocrine factors. Most of these tumors are benign, but some of them do exhibit malignant behavior. Surgery is an appropriate treatment for SCTs-NOS and should be individualized. This study retrospectively analyzed most SCTs-NOS cases published in public journals between 1998 and 2023, and obtained the necessary complete information and prognostic outcomes, which provided effective information and value for the diagnosis and treatment of SCTs-NOS.

## Data availability statement

The raw data supporting the conclusions of this article will be made available by the authors, without undue reservation.

## Ethics statement

The studies involving humans were approved by the Ethical Committee of Tianjin Medical University General Hospital. The studies were conducted in accordance with the local legislation and institutional requirements. The participants provided their written informed consent to participate in this study.

## Author contributions

YS: Project administration, Writing – original draft, Writing – review & editing. LT: Writing – review & editing, Resources. CM: Resources, Writing – review & editing. GL: Funding acquisition, Writing – review & editing.

## References

[1] Hanley KZ Mosunjac MB. Practical review of ovarian sex cord-stromal tumors. Surg Pathol Clin. (2019) 12:587–620. doi: 10.1016/j.path.2019.02.005 31097116

[2] Taylor Hb Fau - Norris HJ Norris HJ. Lipid cell tumors of the ovary. Cancer. (1967) 20:1953–62. doi: 10.1002/1097-0142(196711)20:11<1953::aid-cncr2820201123>3.0.co;2-2 4294036

[3] Tan EA-O Khong CC Bhutia KA-O. A rare case of steroid cell tumor, not otherwise specified (NOS), of the ovary in a young woman. Case rep obstet gynecol. Case Rep Obstet Gynecol. (2019) 2019:4375839. doi: 10.1155/2019/4375839 31428489 PMC6683816

[4] Alves PA-O Sá IA-O Brito M Carnide C Moutinho O. An early diagnosis of an ovarian steroid cell tumor not otherwise specified in a woman. Case Rep Obstet Gynecol. (2019) 2019:2537480. doi: 10.1155/2019/2537480 30792930 PMC6354154

[5] Hayes MC Scully RE. Ovarian steroid cell tumors (not otherwise specified). A clinicopathological analysis of 63 cases. Am J Surg Pathol. (1987) 11:835–45. doi: 10.1097/00000478-198711000-00002 2823622

[6] Wang PH Chao Ht Lee R Lai CR Lai Cr Lee WL. Steroid cell tumors of the ovary: clinical, ultrasonic, and MRI diagnosis–a case report. Eur J Radiol. (1998) 26:269–73. doi: 10.1016/S0720-048X(96)01133-3 9587754

[7] Faraj G Di Gregorio S Misiunas A Faure EN Villabrile P Stringa I. Virilizing ovarian tumor of cell tumor type not otherwise specified: a case report. Gynecol Endocrinol. (1998) 12:347–52. doi: 10.3109/09513599809012837 9859028

[8] Brewer CA Shevlin D. Encouraging response of an advanced steroid-cell tumor to GnRH agonist therapy. Obstet Gynecol. (1998) 92:661–3. doi: 10.1016/s0029-7844(98)00166-5 9764654

[9] Reedy MB Richards WE Ueland F Uy K Lee EY Bryant C. Ovarian steroid cell tumors, not otherwise specified: a case report and literature review. Gynecol Oncol. (1999) 75:293–7. doi: 10.1006/gyno.1999.5549 10525390

[10] Baş F Saka N Darendeliler F Tuzlali S Ilhan R Bundak R. Bilateral ovarian steroid cell tumor in congenital adrenal hyperplasia due to classic 11beta-hydroxylase deficiency. J Pediatr Endocrinol Metab. (2000) 13:663–7. doi: 10.1515/jpem.2000.13.6.663 10905393

[11] Powell JL Dulaney DP Shiro BC. Androgen-secreting steroid cell tumor of the ovary. South Med J. (2000) 93:1201–4. doi: 10.1097/00007611-200093120-00012 11142457

[12] Stephens JW Katz Jr McDermott N MacLean AB Bouloux PMG. An unusual steroid-producing ovarian tumour: case report. Hum Reprod. (2002) 17:1468–71. doi: 10.1093/humrep/17.6.1468 12042263

[13] Garduño-López AL Mondragón-Sánchez R Herrera-Goepfert R Bernal-Maldonado R. Resection of liver metastases from a virilizing steroid (lipoid) cell ovarian tumor. Hepatogastroenterology. (2002) 49:657–9. 12063963

[14] Liu AX Sun J Shao W-Q Shao WQ Jin H-M Song WQ. Steroid cell tumors, not otherwise specified (NOS), in an accessory ovary: a case report and literature review. Gynecol Oncol. (2005) 97:260–2. doi: 10.1016/j.ygyno.2004.12.037 15790472

[15] Kim YT Kim SW Yoon BS Kim SH Kim JH Kim JW. An ovarian steroid cell tumor causing virilization and massive ascites. Yonsei Med J. (2007) 48:142–6. doi: 10.3349/ymj.2007.48.1.142 PMC2628006 17326260

[16] Ding DC Hsu S. Lipid cell tumor in an adolescent girl: a case report. J Reprod Med. (2007) 52:956–8. 17977174

[17] Gupta P Goyal S Gonzalez-Mendoza LE Noviski N Vezmar M Brathwaite CD. Corticotropin-independent cushing syndrome in a child with an ovarian tumor misdiagnosed as nonclassic congenital adrenal hyperplasia. Endocr Pract. (2008) 14:875–9. doi: 10.4158/EP.14.7.875 18996816

[18] Stephens JW Fielding A Verdaguer R Freites O. A steroid-cell tumor of the ovary resulting in massive androgen excess early in the gonadol steroidogenic pathway. Gynecol Endocrinol. (2008) 24:151–3. doi: 10.1080/09513590801917106 18335330

[19] Sawathiparnich P Sitthinamsuwan P Sanpakit K Laohapensang M Chuangsuwanich T. Cushing's syndrome caused by an ACTH-producing ovarian steroid cell tumor, NOS, in a prepubertal girl. Endocrine. (2009) 35:132–5. doi: 10.1007/s12020-009-9150-x 19191036

[20] Lee SH Kang MS Lee GS Chung WY. Refractory hypertension and isosexual pseudoprecocious puberty associated with renin-secreting ovarian steroid cell tumor in a girl. J Korean Med Sci. (2011) 26:836–8. doi: 10.3346/jkms.2011.26.6.836 PMC3102882 21655074

[21] Zhang X Lü B. Ovarian steroid cell tumor, not otherwise specified (NOS): an unusual case with myelolipoma. Int J Gynecol Pathol. (2011) 30:460–5. doi: 10.1097/PGP.0b013e31821643a3 21804391

[22] Varras M Vasilakaki T Skafida E Akrivis C. Clinical, ultrasonographic, computed tomography and histopathological manifestations of ovarian steroid cell tumour, not otherwise specified: our experience of a rare case with female virilisation and review of the literature. Gynecol Endocrinol. (2011) 27:412–8. doi: 10.3109/09513590.2010.495432 20586551

[23] Abdullah L. Ovarian steroid cell tumor, NOS presenting with massive ascites and elevated CA-125. JKAU Med Sci. (2011) 18:107–14. doi: 10.4197/med

[24] Arora R Eble JN Pierce HH Crispen PL DeSimone CP Lee EY. Bilateral ovarian steroid cell tumours and massive macronodular adrenocortical disease in a patient with hereditary leiomyomatosis and renal cell cancer syndrome. Pathology. (2012) 44:360–3. doi: 10.1097/PAT.0b013e328353bf5a 22565324

[25] Singh P Deleon F Anderson R. Steroid cell ovarian neoplasm, not otherwise specified: a case report and review of the literature. Case Rep Obstet Gynecol. (2012) 2012:253152. doi: 10.1155/2012/253152 23091752 PMC3474210

[26] Yılmaz-Ağladıoğlu S Savaş-Erdeve Ş Boduroğlu E Önder A Karaman İ Çetinkaya S. A girl with steroid cell ovarian tumor misdiagnosed as non-classical congenital adrenal hyperplasia. Turk J Pediatr. (2013) 55:443–6. 24292042

[27] Sielert L Liu C Nagarathinam R Craig LB. Androgen-producing steroid cell ovarian tumor in a young woman and subsequent spontaneous pregnancy. J Assist Reprod Genet. (2013) 30:1157–60. doi: 10.1007/s10815-013-0051-9 PMC3800527 23868533

[28] Jiang W Tao X Fang F Zhang S Xu C. Benign and Malignant ovarian steroid cell tumors, not otherwise specified: case studies, comparison, and review of the literature. J Ovarian Res. (2013) 6:53. doi: 10.1186/1757-2215-6-53 23870399 PMC3724598

[29] Swain J Sharma S Prakash V Agrawal NK Singh SK. Steroid cell tumor: a rare cause of hirsutism in a female. Endocrinol Diabetes Metab Case Rep. (2013) 2013:130030. doi: 10.1530/EDM-13-0030 24616767 PMC3922368

[30] Chun YJ Choi Hj Lee HN Cho S Choi JH. An asymptomatic ovarian steroid cell tumor with complete cystic morphology: A case report. Obstet Gynecol Sci. (2013) 56:50–5. doi: 10.5468/OGS.2013.56.1.50 PMC3784108 24327981

[31] Cooray SM Bulugahapitiya UD Samarasinghe K Samarathunga P. Steroid cell tumor not otherwise specified of bilateral ovaries: A rare cause of post menopausal virilization. Indian J Endocrinol Metab. (2013) 17:S262–4. doi: 10.4103/2230-8210.119596 PMC3830327 24251181

[32] Yuan M Qiu M Zhu M. Symptomatic Cushing syndrome and hyperandrogenemia revealing steroid cell ovarian neoplasm with late intra-abdominal metastasis. BMC Endocr Disord. (2014) 14:59–72. doi: 10.1186/1472-6823-14-12 24506845 PMC3930759

[33] Li K Zhu F Xiong J Liu F. A rare occurrence of a Malignant ovarian steroid cell tumor not otherwise specified: A case report and literature review. Oncol Lett. (2014) 8:770–4. doi: 10.3892/ol.2014.2233 PMC4081424 25009655

[34] Tai YJ Chang WC Kuo KT Sheu BC. Ovarian steroid cell tumor, not otherwise specified, with virilization symptoms. Taiwan J Obstet Gynecol. (2014) 53:260–2. doi: 10.1016/j.tjog.2013.04.037 25017282

[35] Chung DH Lee SH Lee KB. A case of ovarian steroid cell tumor, not otherwise specified, treated with surgery and gonadotropin releasing hormone agonist. J Menopausal Med. (2014) 20:39–42. doi: 10.6118/jmm.2014.20.1.39 25371891 PMC4217570

[36] Kim JS Park SN Kim BR. Recurrent ovarian steroid cell tumor, not otherwise specified managed with debulking surgery, radiofrequency ablation, and adjuvant chemotherapy. Obstet Gynecol Sci. (2014) 57:534–8. doi: 10.5468/ogs.2014.57.6.534 PMC4245350 25469345

[37] Haroon S Idrees R Fatima S Memon A Kayani N. Ovarian steroid cell tumor, not otherwise specified: a clinicopathological and immunohistochemical experience of 12 cases. J Obstet Gynaecol Res. (2015) 41:424–31. doi: 10.1111/jog.12537 25345475

[38] Yokozawa T Asano R Nakamura T Furuya M Nagashima Y Koyama-Sato M. Steroid cell tumour, not otherwise specified: Rare case with primary amenorrhoea in a 16-year-old. J Obstet Gynaecol. (2015) 35:867–8. doi: 10.3109/01443615.2015.1022141 26214349

[39] Ben Haj Hassine MA Msakni I Siala H Rachdi R. Laparoscopic management of an ovarian steroid cell tumor, not otherwise specified causing virilization and amenorrhea: A case report. Case Rep Clin Pathol. (2015) 3:10–3. doi: 10.5430/crcp.v3n1p10

[40] Qian L Shen Z Zhang X Wu D Zhou Y. Ovarian steroid cell tumor, not otherwise specified: A case report and literature review. Mol Clin Oncol. (2016) 5:839–41. doi: 10.3892/mco.2016.1071 PMC5228567 28105366

[41] Santo RE Sabino T Agapito A. Ovarian steroid cell tumor not otherwise specified with virilizing manifestations: case report. Acta Obstétrica E Ginecológica Portuguesa. (2016) 10:336–9.

[42] Lee J John VS Liang SX D'Agostino CA Menzin AW. Metastatic Malignant ovarian steroid cell tumor: A case report and review of the literature. Case Rep Obstet Gynecol. (2016) 2016:6184573. doi: 10.1155/2016/6184573 27375912 PMC4916276

[43] Sedhom R Hu S Ohri A Infantino D Lubitz S. Symptomatic Cushing's syndrome and hyperandrogenemia in a steroid cell ovarian neoplasm: a case report. J Med Case Rep. (2016) 10:278. doi: 10.1186/s13256-016-1061-x 27729065 PMC5059940

[44] Zang L Ye M Yang G Li J Liu M Du J. Accessory ovarian steroid cell tumor producing testosterone and cortisol: A case report. Med (Baltimore). (2017) 96:e7998. doi: 10.1097/MD.0000000000007998 PMC5604648 28906379

[45] Chen S Li R Zhang X Lu L Li J Pan H. Combined ovarian and adrenal venous sampling in the localization of adrenocorticotropic hormone-independent ectopic cushing syndrome. J Clin Endocrinol Metab. (2018) 103:803–8. doi: 10.1210/jc.2017-01977 29161416

[46] Yuan X Sun Y Jin Y Chen X Wang X Ji T. Ovarian steroid cell tumor, not otherwise specified, treated with surgery: a case report and review of literature. Int J Clin Exp Pathol. (2019) 12:1434–8. PMC6947056 31933961

[47] Wong FA-O Chan AZ Wong WS Kwan AHW Law TSM Chung JPW. Hyperandrogenism, elevated 17-hydroxyprogesterone and its urinary metabolites in a young woman with ovarian steroid cell tumor, not otherwise specified: case report and review of the literature. Case Rep Endocrinol. (2019) 2019:9237459. doi: 10.1155/2019/9237459 31772787 PMC6854983

[48] Matsukawa J Takahashi T Hada Y Kameda W Ota K Fukase M. Successful laparoscopic resection of virilizing ovarian steroid cell tumor, not otherwise specified, in a 22-year-old woman: a case report and evaluation of the steroidogenic pathway. Fukushima J Med Sci. (2020) 65:133–9. doi: 10.5387/fms.2019-25 PMC7012592 31827013

[49] Nakasone T Nakamoto T Matsuzaki A Nakagami H Aoki Y. Direct evidence on the efficacy of GnRH agonist in recurrent steroid cell tumor-not otherwise specified. Gynecol Oncol Rep. (2019) 29:73–5. doi: 10.1016/j.gore.2019.07.006 PMC6660559 31372485

[50] Yoshimatsu T Nagai K Miyawaki R Moritani K Ohkubo K Kuwabara J. Malignant ovarian steroid cell tumor, not otherwise specified, causes virilization in a 4-year-old girl: A case report and literature review. Case Rep Oncol. (2020) 13:358–64. doi: 10.1159/000506044 PMC7184839 32355490

[51] Faten H Dorra GA-O Slim C Wajdi S Nadia C Kais C. Ovarian steroid cell tumor (Not otherwise specified): A case report of ovarian hyperandrogenism. Case Rep Oncol Med. (2020) 2020:6970823. doi: 10.1155/2020/6970823 32328328 PMC7168706

[52] Uyanıkoglu H Ozer G Kahraman S. A spontaneous pregnancy and live birth in a woman with primary infertility following the excision of an ovarian adrenal rest tumor: A rare case. Clin Exp Reprod Med. (2020) 47:319–22. doi: 10.5653/cerm.2020.03692 PMC7711094 33105528

[53] Schnuckle EM Williamson A Carpentieri D Taylor S. Ovarian sex cord stromal tumor, steroid cell, NOS in an adolescent: A case report. J Pediatr Adolesc Gynecol. (2021) 34:94–7. doi: 10.1016/j.jpag.2020.08.001 32781238

[54] Ismail SA-O Hraib M Issa R Alassi T Alshehabi Z. A large ovarian steroid cell tumor-not otherwise specified with a unique combination of benign and Malignant features as a challenging cause of oligomenorrhea and hirsutism in a 21-year-old Syrian female: a case report. BMC Womens Health. (2021) 21:95. doi: 10.1186/s12905-021-01244-1 33663470 PMC7934245

[55] Lin M Bao K Lu L Xu S Liang Y Cheng X. Ovarian steroid cell tumors, not otherwise specified: analysis of nine cases with a literature review. BMC Endocr Disord. (2022) 22:265. doi: 10.1186/s12902-022-01170-9 36316664 PMC9623933

[56] Matemanosak PA-O Peeyananjarassri KA-O Suwanrath CA-O Wattanakumtornkul S Klangsin SA-O Thiangphak EA-O. Ovarian steroid cell tumor (not otherwise specified) with subsequent spontaneous pregnancy after tumor removal: a case report and literature review. Gynecol Endocrinol. (2023) 39:2186138. doi: 10.1080/09513590.2023.2186138 36878245

[57] Luk Wt Lee N Chang TC Chu KK. Lipid cell tumor of the ovary associated with endometrial adenocarcinoma–a case report. Changgeng Yi Xue Za Zhi. (1989) 12:244–8. 2637062

[58] Elhadd TA Connolly V Cruickshank D Kelly WF. An ovarian lipid cell tumour causing virilization and Cushing's syndrome. Clin Endocrinol (Oxf). (1996) 44:723–5. doi: 10.1046/j.1365-2265.1996.693515.x 8759186

[59] Donovan JT Otis Cn Powell JL Cathcart HK. Cushing's syndrome secondary to Malignant lipoid cell tumor of the ovary. Gynecol Oncol. (1993) 50:249–53. doi: 10.1006/gyno.1993.1202 8375740

[60] Anderson PW d'Ablaing G 3rd Penny R Sherrod A Do YS. Secretion of prorenin by a virilizing ovarian tumor. Gynecol Oncol. (1992) 45:58–61. doi: 10.1016/0090-8258(92)90491-Z 1318255

[61] Ghose AA-O McCann L Makker SA-O Mukherjee U Gullapalli SVN Erekkath J. Diagnostic biomarkers in ovarian cancer: advances beyond CA125 and HE4. Ther Adv Med Oncol. (2024) 16:17588359241233225. doi: 10.1177/17588359241233225 38435431 PMC10908239

[62] Zhu X Zhou L Jiang L. Steroid cell tumours: rare ovarian tumours that cause hyperandrogenaemia in postmenopausal women(2020). Available online at: https://doiorg/1021203/rs3rs-136187/v1. doi: 10.21203/rs.3.rs-136187/v1

[63] Saida T Tanaka Yo Minami M. Steroid cell tumor of the ovary, not otherwise specified: CT and MR findings. AJR Am J Roentgenol. (2007) 188:W393–4. doi: 10.2214/AJR.06.0867 17377014

[64] Bourgioti C Konidari MA-O Moulopoulos LA-O. Manifestations of ovarian cancer in relation to other pelvic diseases by MRI. Cancers (Basel). (2023) 15:2106. doi: 10.3390/cancers15072106 37046767 PMC10093428

[65] Jung SE Lee JM Rha SE Byun JY Jung JI Hahn ST. CT and MR imaging of ovarian tumors with emphasis on differential diagnosis. Radiographics. (2002) 22:1305–25. doi: 10.1148/rg.226025033 12432104

[66] Jung SE Rha SE Lee JM Park SY Oh SN Cho KS. CT and MRI findings of sex cord-stromal tumor of the ovary. AJR Am J Roentgenol. (2005) 185:207–15. doi: :10.2214/ajr.185.1.01850207 15972425

[67] Pinto AA-O Martins MB Oliveira N Oliveira M. Ovarian steroid cell tumour inducing virilisation in a postmenopausal woman. BMJ Case Rep. (2022) 15:e249907. doi: 10.1136/bcr-2022-249907 PMC9021769 35444026

[68] Mehdi G Ansari HA Sherwani RK Rahman K Akhtar N. Ovarian steroid cell tumour: correlation of histopathology with clinicopathologic features. Patholog Res Int. (2011) 2011:987895. doi: 10.4061/2011/987895 21436872 PMC3049327

[69] Bhagat R Bodal VK Gupta N Garg P. Steroid cell tumour of ovary - A rare case report. J Clin Diagn Res. (2016) 10:ED06–ED7. doi: 10.7860/JCDR/2016/15767.8556 PMC5071945 27790445

[70] Murhekar K Louis R Majhi U Majhi U. A rare occurrence of a steroid cell tumor of the pelvic mesentery: a case report. J Med Case Rep. (2011) 5:517–21. doi: 10.1186/1752-1947-5-517 PMC3212982 22008415

[71] Roth LM. Recent advances in the pathology and classification of ovarian sex cord-stromal tumors. Int J Gynecol Pathol. (2006) 25:199–215. doi: 10.1097/01.pgp.0000192271.22289.e6 16810055

[72] Gershenson DM Copeland LJ Kavanagh JJ Stringer CA Saul PB Wharton JT. Treatment of metastatic stromal tumors of the ovary with cisplatin, doxorubicin, and cyclophosphamide. Obstet Gynecol. (1987) 70:765–9. 3658288

[73] Homesley HD Bundy Bn Fau - Hurteau JA Hurteau Ja Fau - Roth LM Roth LM. Bleomycin, etoposide, and cisplatin combination therapy of ovarian granulosa cell tumors and other stromal Malignancies: A Gynecologic Oncology Group study. Gynecol Oncol. (1999) 72:131–7. doi: 10.1006/gyno.1998.5304 10021290

[74] Pecorelli S Wagenaar HC Vergote IB Curran D Beex LV Wiltshaw E. Cisplatin (P), vinblastine (V) and bleomycin (B) combination chemotherapy in recurrent or advanced granulosa(-theca) cell tumours of the ovary. An EORTC Gynaecological Cancer Cooperative Group study. Eur J Cancer. (1999) 35:1331–7. doi: 10.1016/S0959-8049(99)00142-2 10658523

[75] Brown J Shvartsman HS Deavers MT Ramondetta LM Burke TW Munsell MF. The activity of taxanes compared with bleomycin, etoposide, and cisplatin in the treatment of sex cord-stromal ovarian tumors. Gynecol Oncol. (2005) 97:489–96. doi: 10.1016/j.ygyno.2005.01.011 15863149

[76] Pascale MM Pugeat M Roberts M Rousset H Déchaud H Dutrieux-Berger N. Androgen suppressive effect of GnRH agonist in ovarian hyperthecosis and virilizing tumours. Clin Endocrinol (Oxf). (1994) 41:571–6. doi: 10.1111/j.1365-2265.1994.tb01820.x 7828344

[77] Wang PH Chao Ht Lee WL. Use of a long-acting gonadotropin-releasing hormone agonist for treatment of steroid cell tumors of the ovary. Fertil Steril. (1998) 69:353–5. doi: 10.1016/s0015-0282(97)00500-1 9496356

[78] Vasilevska DA-O Rudaitis V Vasilevska D Mickys UA-O Wawrysiuk S Semczuk AA-O. Failure of multiple surgical procedures and adjuvant chemotherapy in early-stage steroid-cell ovarian tumor treatment: a case report and literature review. J Int Med Res. (2021) 49:300060520983195. doi: 10.1177/0300060520983195 33435776 PMC7809311

